# Identification of metabolism-associated molecular classification and prognostic genes for medulloblastoma based on bioinformatics analysis

**DOI:** 10.1016/j.clinsp.2026.101003

**Published:** 2026-05-18

**Authors:** Zihan Yan, Yunwei Ou, Xu Han, Jian Gong

**Affiliations:** a Department of Pediatric Neurosurgery, Beijing Tiantan Hospital, Capital Medical University, Beijing, China; b Department of Neurosurgery, Beijing Neurosurgical Institute, Beijing, China; c Institute of Artificial Intelligence, Hefei Comprehensive National Science Center, Hefei City, Anhui Province, China

**Keywords:** Medulloblastoma, Solid brain tumor, Bioinformatics analysis, Metabolism, Gene

## Abstract

**Objective:**

Medulloblastoma (MB) is one of the most prevalent solid brain tumors in the pediatric population. Although hundreds of Differentially Expressed Genes (DEGs) have been identified as diagnostic molecules and therapeutic targets for Medulloblastoma (MB), the function of metabolism-associated genes in the pathophysiological mechanisms of MB remains unclear.

**Methods:**

The Gene Expression Omnibus (GEO) provided five datasets that contain mRNA expression profiles and clinical information. In detail, 599 MB samples extracted from GSE85217 were set as the training set (*n* = 400) and test set (*n* = 199). For validation, 74 samples from GSE37418, 8 scRNA-seq samples, and 37 RNA-seq samples were used. Nonnegative matrix factorization clustering was conducted, leading to the identification of four MB subclasses (C1, C2, C3, and C4) in both the training and test sets.

**Results:**

C1 and C3 are metabolically active, and C2 involves metabolic hypoactivity; C4 did not exhibit obvious metabolic characteristics. Next, GSE50161, GSE74195, and GSE86574 were used to identify DEGs, and a 17-gene metabolism-associated signature for prognosis prediction was established. The authors performed transcriptome and single-cell sequencing on medulloblastoma bulk specimens to further validate metabolic subclasses and the prognosis prediction model at the transcriptome and cellular level.

**Conclusion:**

The present study classifies Medulloblastoma (MB) based on metabolic signatures, supplementing existing subtype characterization from a metabolic perspective. The authors provide preliminary insights into MB’s metabolic hallmarks and a potential reference for developing multimolecule-based personalized therapies and prognostic tools ‒ with the caveat that the 17-gene signature requires additional multi-center validation to confirm clinical utility.

## Highlights

• Identified four Medulloblastoma (MB) subclasses via clustering.
• Established a 17-gene metabolism-associated signature for prognosis prediction.
• Validate metabolic subclasses and prognosis model with transcriptome and single-cell data.

## Introduction

Medulloblastoma (MB) stands out as a prevalent solid brain tumor in the pediatric population, making up 15 %‒20 % of central nervous system tumors in children.^1^ Epidemiological investigations indicate that MB frequently occurs in children aged 0 to 9, with a male predominance.^2^ The World Health Organization (2016) designates MB as a grade IV tumor and further divides it into four molecular subgroups: the WNT, SHH, Group 3, and Group 4.^3^ Each of these subtypes possesses distinct epidemiological and molecular traits.^4^ This classification is recognized as an important basis for the clinical evaluation of MB. Clinically, Medulloblastoma (MB) primarily arises in the posterior fossa, presenting with increased intracranial pressure, and tends to metastasize via the cerebrospinal fluid at early stages of the disease course.^5^ Current treatment modalities include maximal safe surgical resection, radiotherapy, and chemotherapy.^5^ Nevertheless, one-third of patients ultimately succumb to the disease. Overall, targeted therapy is regarded as the optimal treatment strategy.

Metabolic reprogramming is acknowledged as one of the hallmarks of cancer.^6^ and concentrating on the metabolic differences between tumor cells and normal cells offers a promising anticancer strategy.^7^ Up to now, multiple studies have probed into the gene expression profiles of MB and pinpointed hundreds of DEGs as potential diagnostic markers and therapeutic targets.^8^, ^9^, ^10^, ^11^, ^12^ However, the function of metabolism-associated genes in the pathophysiological processes of MB remains elusive.

Within the scope of this study, the authors applied NMF clustering based on metabolic genes to MB datasets from GEO, validated the results, and identified four distinct subclasses. The authors further characterized their prognostic traits and metabolic signatures, developed a metabolism-associated gene signature, and constructed an integrated nomogram incorporating clinical factors and the 17-gene signature. These findings shed light on the metabolic gene expression landscape of MB, offering potential novel therapeutic targets for clinical intervention.

## Materials and methods

### Data acquisition and processing

The gene expression profiles and platform annotations of MB tissues and healthy brain tissues were retrieved from the GEO database, including datasets GSE37418, GSE50161, GSE74195, GSE85217, and GSE86574 (Supplementary Table 1). After data processing, 660 MB samples and 28 samples of healthy brain tissues were incorporated into the analysis. GSE85217, which contained 599 MB samples with complete survival and clinical data, was randomly stratified by subgroup and survival status (deceased, alive, or censored) into an exploratory cohort (400 samples) and an internal validation cohort (199 samples).

For external validation, 45 Chinese patients were recruited from Tiantan Hospital, Capital Medical University, during the period from September 2016 to November 2022. This study was conducted in accordance with the Declaration of Helsinki. All participants offered informed consent. which was ratified by the Institutional Review Board (IRB) of Beijing Tiantan Hospital, Capital Medical University (n°KY2022–082–01). This study follows the STROBE Statement. Among them, 37 underwent transcriptome sequencing (RNA-seq), while 8 underwent single-cell RNA sequencing (scRNA-seq). All MB diagnoses were pathologically confirmed. Tissue specimens were stored in liquid nitrogen within 5-minutes of resection to preserve integrity.

### Identification of MB subclasses

The authors analyzed 2503 metabolism-associated genes involved in global metabolic processes using NMF clustering,^13^ with preprocessing steps to exclude genes with zero expression in any sample or a Median Absolute Deviation (MAD) < 0.5 across all samples. Unsupervised NMF clustering was performed on both training and test sets using the NMF R package (v0.26). Principal Component Analysis (PCA) was utilized to assess transcriptional variations among subclasses.

### RNA sequencing and data analysis of MB tissues

Total RNA was extracted from fresh frozen tissues with the TRIzol reagent. Integrity was appraised by using the RNA Nano 6000 Assay Kit with the Agilent 2100 Bioanalyzer (Agilent Technologies, CA, USA). Libraries were prepared with the TruSeq PE Cluster Kit v3-cBot-HS (Illumina) in accordance with manufacturer protocols and sequenced on the Illumina HiSeq platform. Raw fastq data were handled using Perl (v5.36.0) scripts to eliminate adapter sequences, Poly-N reads, and low-quality reads, thus obtaining clean data. Q20, Q30, and GC content were computed for quality control. Data were further processed using RSEM v1.3.0, with the GRCh38 RefSeq genome serving as the reference. Transcripts with |log2(fold change)| > 1 and *p* < 0.05 were identified as DEGs.

### ScRNA sequencing and data analysis of MB tissues

For the Chromium Single Cell Gene Expression Flex (scFFPE-seq) procedure, 25 μm FFPE curls were gathered into a tube before being serially sectioned for Visium CytAssist and Xenium. After that, an extra 25 μm FFPE curl was put into the same tube, which was set aside for scFFPE-seq. These combined 25 μm curls were considered a single biological replicate. Because scFFPE-seq needs a large quantity of input material and the same tissue block has to be kept for multiple technical analyses, a second replicate couldn't be created.

After isolating cells, 10,000 cells or nuclei underwent barcoding and library construction. The libraries were sequenced on the Illumina NovaSeq 6000 platform to get 50,000 reads per cell. Raw sequencing data were processed using CellRanger (v6.1.2, 10 × Genomics) and Seurat (v3.1.0). Cells were filtered using the criteria of nFeature_RNA ranging from 200 to 6000, nCount_RNA greater than 500, and percent.mt <10 %; dimensionality reduction was performed via RunPCA with features set as VariableFeatures(object = seurat_obj) and npcs = 50; clustering was conducted using FindNeighbors with dims = 1:30 followed by FindClusters with a resolution of 0.5 (the optimal resolution determined by elbow plot and silhouette score); UMAP visualization was implemented with RunUMAP using dims = 1:30, n.neighbors = 30, and min.dist = 0.3, while Copy Number Variations (CNVs) were detected with CopyKat (v.1.1.0). DEG analysis and marker gene identification were carried out using the FindAllMarkers function in Seurat, with the “only.pos” parameter set to TRUE to keep only genes with notably higher expression in specific clusters. The Wilcoxon test was applied to each gene (inclusion criteria: detection in at least 25 % of cells in either of the two populations), and both p-values and adjusted p-values were calculated to assess statistical significance.

### Transcriptome data analysis

For the GEO medulloblastoma dataset, the authors performed enrichment analysis on medulloblastoma metabolic subgroups using Gene Set Variation Analysis (GSVA).^14^ To perform RMA (Robust Multi-array Average) normalization on microarray data and remove batch effects, the authors employed a workflow combining the limma R package (v3.56.2) with the SVA R package (v3.48.0) (for batch effect correction). Meanwhile, the authors conducted differential analysis of core signature genes across multiple datasets with the limma R package. DEG meeting the criteria of absolute p-value < 0.05 and |log2 (fold change)| > 1 were included in subsequent studies, and Gene Ontology (GO) and KEGG pathway enrichment analyses were performed on them via the WebGestalt platform (http://webgestalt.org/option.php). In addition, overall survival (OS)-related genes were identified through the Cox proportional hazards model, and univariate analyses were conducted using the survival R package (v3.5–7) to filter out genes associated with OS.

### Metabolism-associated signature construction model

The prognostic gene signature, presented as a risk score, was constructed using Lasso-penalized Cox regression analysis implemented via the *R* package glmnet (v4.1–8). Prior to model fitting, gene expression data were automatically *Z*-score standardized by glmnet to eliminate dimensional differences among genes, ensuring equitable application of the penalty across all variables. Patients were stratified into high-risk and low-risk groups using the median risk score as the cutoff. Kaplan-Meier (KM) survival curves and time-dependent Receiver Operating Characteristic (ROC) curve analyses were subsequently performed to evaluate the predictive performance of the model.

### Gene signature-based prognostic model as an independent predictor of overall survival

Univariate and multivariate Cox regression analyses were applied to determine whether the prognostic model could serve as an independent predictor of OS in MB patients, irrespective of other clinicopathological factors. Clinical characteristics were treated as independent variables, with OS as the dependent variable, to compute the Hazard Ratio (HR), 95 % Confidence Interval (95 % CI), and two-sided p-value.

### Bootstrap validation for model stability

Ordinary nonparametric bootstrapping (1000 iterations of resampling with replacement) was performed in R 4.4.2 to evaluate model stability and overfitting risk. Each iteration resampled 400 samples from the discovery cohort. Time-dependent AUC values for 1-, 3-, and 5-year Overall Survival (OS) were calculated via the timeROC package (v0.4–3, weighting = “marginal”, cause = 2, id = TRUE). The boot package (v1.3–28) implemented resampling, and mean AUC ± Standard Deviation (SD) was summarized.

### Construction of a predictive nomogram

A prognostic nomogram was constructed using a stepwise Cox regression model to predict the 1-, 3-, and 5-year OS of MB patients, with subsequent ROC analysis performed. This model was further validated in the validation cohort.

### Cell function experiments

Lentivirus Packaging and Detection: Key reagents and consumables included DH5α/Stbl3 competent cells, 2 × GPV8 PCR Master Mix, lentiviral vectors, restriction enzymes, DNA ligase, and sequencing primers, all purchased from specified suppliers with respective catalog numbers. Instruments used were carbon dioxide incubators (Thermo Fisher Scientific, model 3111), biosafety cabinets (Labconco, model 3620), PCR thermocyclers (Bio-Rad, model T100), ultracentrifuges (Beckman Coulter, model Optima l-100 K), and inverted microscopes (Olympus, model IX73). Lentiviral plasmids were constructed via restriction enzyme digestion and target fragment recovery. The target fragment was amplified by PCR, ligated with the linearized vector, and transformed into DH5α/Stbl3 competent cells. Positive clones were identified by bacterial solution PCR and confirmed by first-generation sequencing. Lentivirus packaging was performed using 293T-cells transfected with the recombinant plasmid and packaging vectors. Viral supernatants were collected at 48- and 72-hours post-transfection, concentrated via ultracentrifugation (25,000 × *g* for 2-hours at 4 °C), and stored at −80 °C. Virus stocks were tested for sterility (culture-based method), mycoplasma (PCR assay), and titer (serial dilution method with 293T-cells).

Reagents and consumables included Trypan Blue Staining Reagent (Sigma-Aldrich, catalog n° T8154), CCK-8 Kit (Dojindo, catalog n° CK04), and 96-well cell culture plates (Corning, catalog n°3599). Instruments used were a hemocytometer (Neubauer, model 717,840), carbon dioxide incubator (Thermo Fisher Scientific, model 3111), and microplate reader (Bio-Rad, model iMark). Cryopreserved cells were thawed in a 37 °C water bath, disinfected with 75 % ethanol, and cultured in complete medium. Cells were passaged when confluency reached 80 %‒90 %, counted using Trypan Blue to ensure > 90 % viability, and seeded into 96-well plates at 3 × 10^3^ cells/well. After 24-hours of adhesion, 10 μL CCK-8 working solution was added to each well, followed by incubation at 37 °C for 2-hours. Absorbance at 450 nm was measured daily for 5 consecutive days.

Reagents included serum-free medium, complete medium (10 % FBS), and Matrigel (Corning, catalog n°356,234). Consumables were 24-well Transwell chambers with 8 μm pores (Corning, catalog n° 3422) and crystal violet staining solution (Sigma-Aldrich, catalog n° C3886). Instruments used were a centrifuge (Eppendorf, model 5810R), carbon dioxide incubator (Thermo Fisher Scientific, model 3111), and an inverted microscope (Olympus, model IX73). In migration assays, 200 μL of the cell suspension was placed in the upper chamber, while 600 μL of complete medium was added to the lower chamber. For invasion assays, Matrigel (1:8 dilution) was pre-coated onto the upper chamber membrane and polymerized at 37 °C for 30-minutes before adding cells. After a 24-hour incubation period, cells were fixed with 4 % paraformaldehyde, stained with crystal violet, washed, air-dried, and imaged under a microscope. Five random fields of view were selected for quantification and counting.

### Statistical analysis

All statistical analyses were performed using R 4.4.2. Unless otherwise specified, p-value < 0.05 was deemed statistically significant.

## Results

### NMF identifies four metabolism subclasses in MB

**Fig. 1:**
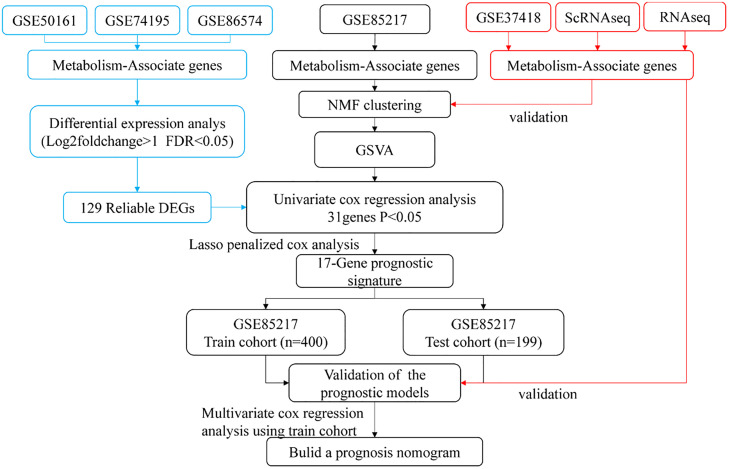
Flowchart of this study.

The workflow is presented in Fig. 1. Table 1 summarizes the clinical characteristics of the training and test sets, which were similar except for age distribution. The training set (GSE85217) included 599 valid MB samples, all with complete clinical data. For clustering analysis, 2752 initially identified metabolism-associated genes in the training set were acquired. Following preliminary filtering, 249 genes were removed due to undetectable expression or low median absolute deviation, leaving 2503 genes for subsequent analyses.

**Table 1:** The clinical characteristics of the training and test sets.

Clinical characteristics	Training set (*n* = 599)	Testing set (*n* = 74)	RNAseq set (*n* = 37)	ScRNAseq set (*n* = 8)	p-value
Gender					0.087
male	292	52	27	4	
female	207	22	10	4	
Age					0.024
< 10	370	55	30	5	
> 10	229	19	7	3	
WNT	60 (10.0 %)	8 (10.8 %)	5 (13.5 %)	1 (12.5 %)	0.353
SHH	168 (28.0 %)	11 (14.9 %)	5 (13.5 %)	3 (37.5 %)	
Group3	111 (18.5 %)	16 (21.6 %)	8 (21.6 %)	1 (12.5 %)	
Group4	260 (43.4 %)	39 (52.7 %)	19 (51.4 %)	3 (37.5 %)	

**Fig. 2:**
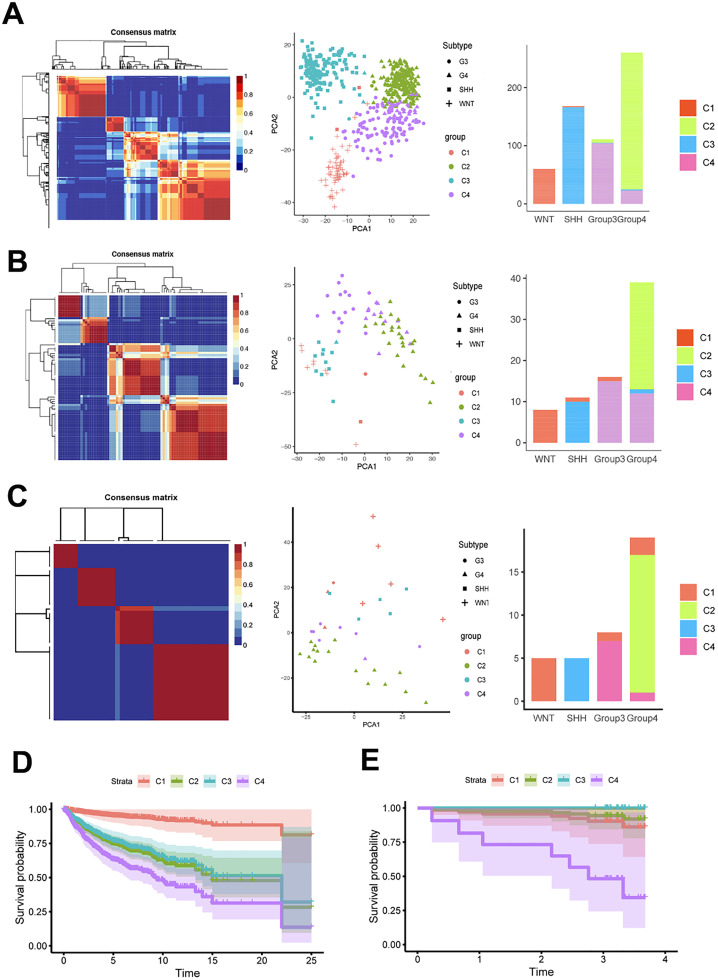
Identification of MB subclasses using NMF consensus clustering. NMF Clustering Using Metabolism-Associated Genes to Uncover Metabolic clusters in training (A) test (B) and validation (C) set. PCA revealed the distribution patterns of the four MB subclasses across the training (A) test (B) and validation (C) set. Stacked histogram showed the distribution of four MB subclasses in training (A) test (B) and validation (C) set. OS of four subclasses (C1, C2, C3 and C4) in training set (D) and validation (E) set.

Cophenetic correlation coefficients were computed to identify the optimal κ-value, with a sharp drop observed when κ = 4. The consensus matrix heatmap clearly showed distinct boundaries at κ = 4 (Fig. 2A). Thus, κ = 4 was chosen as the optimal number of clusters, resulting in four clusters in the training set: 62 samples in C1, 241 in C2, 169 in C3, and 127 in C4.

The authors then performed Principal Component Analysis (PCA) to assess subclass assignments, revealing four clusters distinctly positioned within the two-dimensional coordinate system. The stacked histogram depicts the distribution of the four MB subclasses within the training set (Fig. 2A). In addition, the authors extracted 74 eligible MB samples from the GEO database (GSE37418) and expression data for the same genes selected above in the test set. A parallel NMF consensus clustering analysis was performed on the test set, which consistently identified four as the optimal κ-value-mirroring the clustering distribution pattern observed in the training set (Fig. 2B). In the RNA-seq validation set, the authors can observe that despite the different detection methods, the results are consistent with the previous two sets. The NFM clustering can still be divided into four metabolic clusters (Fig. 2C and Supplementary Table 2).

### Metabolic genes are associated with MB subgroups and prognosis

To determine the relationship among the clinical features of each MB subclass, the chi-square test and Bartlett test were performed. The results for the training set, presented in Table 2, reveal significant variations in the distribution of the WNT, SHH, Group 3, and Group 4 subgroups across the four clusters. Furthermore, KM survival curves revealed significant differences existed among the C1 – 4 groups (Fig. 2D and E; training set, *p* < 0.001; validation set, *p* = 0.013). Although the p-values of regression curves based on molecular typing were comparable to those of metabolic subclasses in both the training and testing sets, metabolism-associated genes still hold significant importance for the classification of MB subgroups and the prediction of patient prognosis.

**Table 2:** Clinical Characteristics of patients with distinct classification in training set.

Clinical characteristics	Total	C1	C2	C3	C4	p-value
	*n* = 599	*n* = 62	*n* = 241	*n* = 169	*n* = 127	
Gender						0.001223
Male		28	166	106	92	
Female		34	75	63	35	
Age		13.21±9.60	9.14±5.24	12.95±13.16	7.28±6.80	*p* < 0.001
< 10		25	147	95	103	
> 10		37	94	74	24	
Subgroup						*p* < 0.001
WNT		61	0	0	0	
SHH		1	0	166	0	
Group3		0	6	1	104	
Group4		0	235	2	23	

### ScRNA-seq analysis validates metabolic subcluster and their characteristics at the level of MB tumor cells

**Fig. 3:**
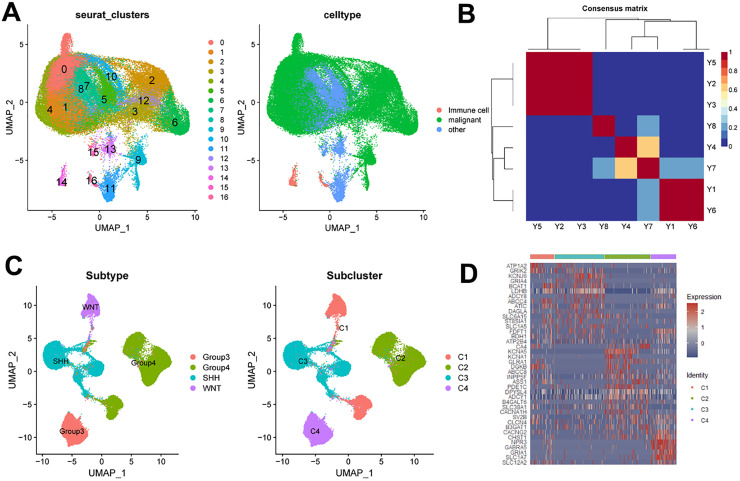
ScRNA-seq analysis validates metabolic subcluster at the level of MB Tumor Cells. (A) UMAP projection of 62,428 single cells from 8 patients shows 17 clustering. The number of malignant cells was 49,879 cells, immune cells was 907 cells, and the number of other cells, including oligodendrocytes, astrocytes, and microglia, totaled was 11,642 cells; (B) Validate the previously identified metabolic subgroups; (C) The UMAP of different molecular subtypes and metabolic subcluster in malignant cells; (D) DEG heatmap of metabolic cluster in malignant cells.

To explore the metabolic subcluster at the tumor cell level of MB, the authors first obtained FFPE tissues from 8 MB patients and conducted scRNA-seq analysis using the 10 × Chromium technology (Supplementary Table 3). The authors obtained a total of 62,428 single cells across 17 clusters. CNVs were identified using the CopyKat software. Leveraging these identified CNVs, a clear distinction could be made between tumor cells and normal cells. Specifically, the number of malignant cells was 49,879 cells, the number of immune cells was 907 cells, and the number of other cells, including oligodendrocytes, astrocytes, and microglia, totaled 11,642 cells (Fig. 3A and Supplementary Table 4). Subsequently, the authors calculated the average gene expression level of each malignant cell to validate the previously identified metabolic subgroups (Fig. 3B). Reclustering of malignant cells based on metabolic genes revealed that cells from different molecular subtypes and metabolic subclasses clustered together, consistent with our tissue-level findings (Fig. 3C and D).

### MB subclass metabolism signatures

**Fig. 4:**
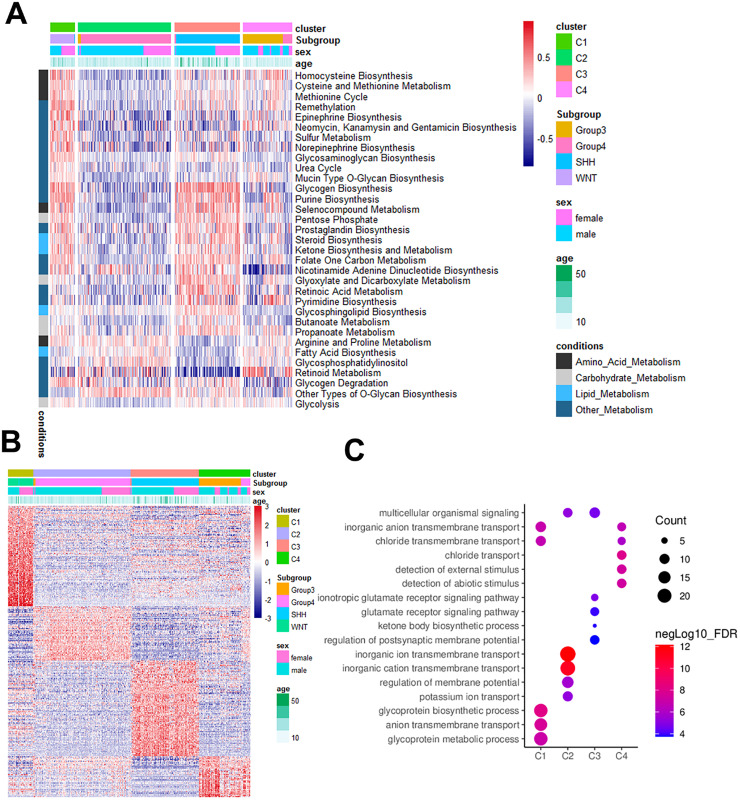
Metabolic differences among medulloblastoma subclasses. (A) Heatmap of GSVA-differentiated pathways in the training set; (B) Heatmap of subclasses differentiated genes in the training set; (C) GO enrichment results of subclasses differentiated genes in the training set.

The authors further evaluated whether different clusters have different metabolic characteristics to assess the metabolic characteristics of MB clusters. The authors first identified and quantified 114 metabolic processes using the GSVA R package. Each sample was assigned a score corresponding to a specific metabolic pathway, following which differential analyses were conducted to unravel subtype-specific metabolic traits (Supplementary Table 5). The results showed 22 significantly different metabolic pathways for C1 compared with the other clusters, of which 21 were upregulated; four are related to amino acid metabolism. For C2, the authors identified 18 differential metabolic pathways, 17 of which were downregulated, encompassing amino acids, carbohydrates, lipids, and other metabolic features. There were 19 different metabolic pathways for C3; 15 were upregulated, and 4 were related to carbohydrate metabolism. In contrast, only one significantly different metabolic pathway was observed for C4. After merging repetitive pathways in the four clusters, a heatmap showed 33 metabolic-related features (Fig. 4A). C1 and C3 are related to metabolic activity and C2 to metabolic hypoactivity, whereas C4 does not display obvious metabolic characteristics. The aforementioned data indicate that these four subclasses are enriched in distinct metabolic pathways and exhibit varying levels of metabolic activity. Overlapping pathways across subclasses were counted once, resulting in 33 unique pathways for the heatmap.

### DEGs of MB subclasses

To further explore the molecular features of the four metabolism-associated MB subclasses, DEGs were identified in the dataset, and GO analysis was performed (Figs. 4B and C). Using thresholds of |log2 FC| > 1 and adjusted *p* < 0.05, a total of 382 upregulated DEGs were detected across the four subclasses. Specifically, 132 DEGs were identified in C1, 70 in C2, 118 in C3, and 48 in C4 (Supplementary Table 6). Following the merger, 345 genes were obtained (Fig. 4B). Similarly, consistent characteristics were observed in the differential gene analysis of single-cell metabolic subclusters (Fig. 3D and Supplementary Table 7). GO analysis revealed that these genes were enriched in distinct pathways, indicating that the four subclasses possess unique molecular signatures (Fig. 4C).

### Identification of DGEs and survival-related genes

**Fig. 5:**
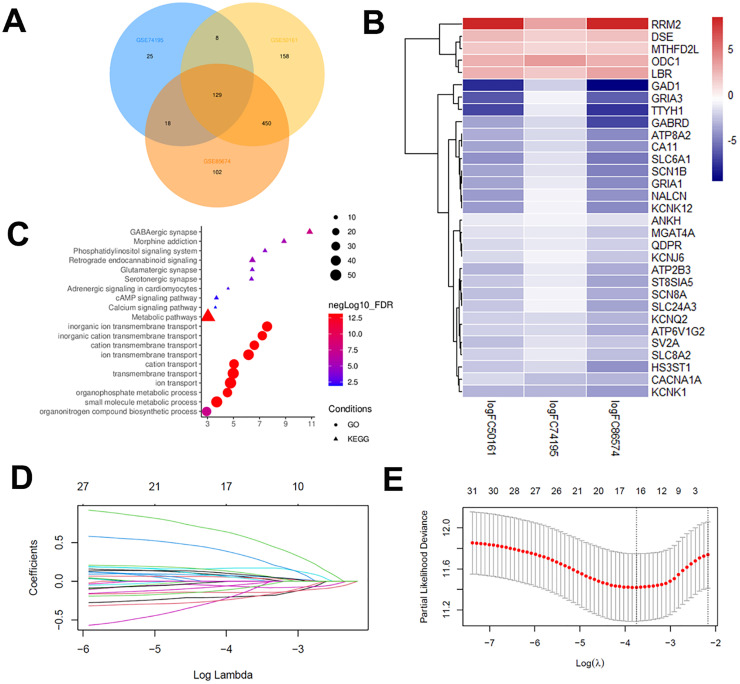
Identification of a metabolism-associated signature using the LASSO regression model. (A) Venn diagram of differentially expressed genes across the mRNA expression profiling datasets GSE74195, GSE50161, and GSE86574; (B) The heatmap of hub Genes; (C) GO and KEGG enrichment of hub Genes; (D) Filtration of variables in LASSO regression; (E) 17-genes were obtained from LASSO regression.

DEGs (745 in GSE50161, 180 in GSE74195 and 699 in GSE86574) were identified by standardizing microarray results and extracting metabolism-associated genes. The intersection of the datasets resulted in 129 DEGs, as illustrated in the Venn diagram (Supplementary Table 8 and Fig. 5A), comprising 95 downregulated and 34 upregulated genes when comparing MB tissues with healthy brain tissues. The authors applied a univariate Cox proportional hazards model analysis to the GSE85217 dataset to identify metabolic genes with prognostic significance. Among the aforementioned metabolic genes, only 31 showed a significant association with OS (Fig. 5B and Supplementary Table 9). Functional enrichment analysis results for these DEGs are presented in Fig. 5C. GO analysis indicated that the DEGs are significantly enriched in pathways including ion transport, small molecule metabolic processes, organophosphate metabolic processes, and organonitrogen compound biosynthesis; KEGG analysis indicated enrichment in metabolic pathways, phosphatidylinositol signaling system, and glutamatergic synapse (Fig. 5C).

### Establishment a 17-gene prognostic signature

The GSE85217 dataset was divided into a training group (400 samples) and a test group (199 samples) (Supplementary Table 10). Lasso penalized Cox analysis in the training group identified 17 genes to construct the prognostic model, including MTHFD2L, QDPR, ODC1, GRIA3, TTYH1, RRM2, ATP2B3, HS3ST1, GRIA1, KCNK1, NALCN, GAD1, ATP6V1G2, ATP8A2, DSE, SLC24A3, and ANKH (Fig. 5D‒E and Supplementary Table 11). The risk score was determined using the following formula: 0.6616 × MTHFD2L (expression) + 0.413 × QDPR + 0.165 × ODC1 + 0.143 × GRIA3 + 0.112 × TTYH1 + 0.085 × RRM2 + 0.075 × ATP2B3 + 0.071 × HS3ST1 + 0.057 × GRIA1 - 0.010 × KCNK1 - 0.056 × NALCN - 0.070 × GAD1 - 0.123 × ATP6V1G2 - 0.144 × ATP8A2 - 0.208 × DSE - 0.252 × SLC24A3 - 0.254 × ANKH. The authors then validated the prognostic predictive capacity of this 17-gene signature in the test set to confirm our findings from the training set (Fig. 5).

### Kaplan-Meier survival curves analysis of the 17-gene signature

**Fig. 6:**
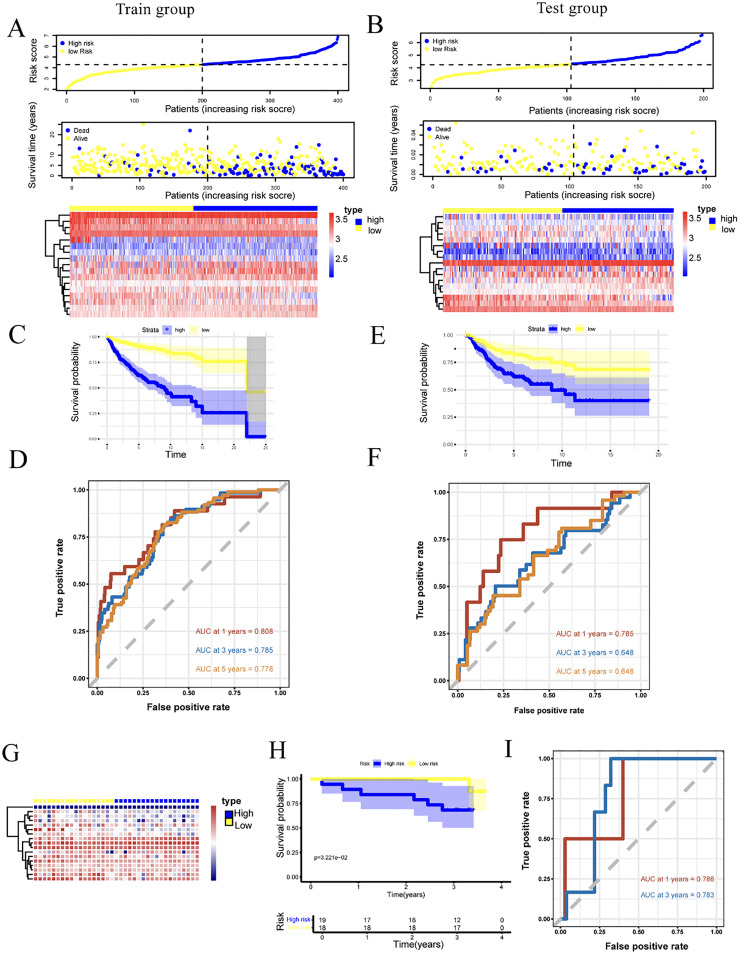
Development and validation of prognostic signature based on 17-Gene in training and testing cohorts. (A‒B) The risk and survival distribution of the patients, and the heatmap of the 17-genes in training and testing cohorts; (C‒D) KM curve revealing the significantly shorter OS in the high-risk group compared with the low-risk group in training and testing cohorts; (E‒F) ROC curve of the risk signature in training and testing cohorts; (G) Heatmap of the 17-genes in validation cohorts; (H) KM curve revealing the significantly shorter OS in the high-risk group compared with the low-risk group in validation cohorts; (I) ROC curve of the risk signature in validation cohorts.

The authors used the KM survival curve analysis to compare the overall survival between the two groups divided based on the median risk score. Through the analysis of survival status and 17-gene expression profiles in high-risk and low-risk groups, the authors found that the high-risk group had a worse prognosis (Figs. 6A‒B). Additionally, the prognostic predictive performance of gene signatures was assessed using the time-dependent ROC curves.

In the training cohort, OS significantly differed between the high-risk and low-risk groups (*p* < 0.001), with the 17-gene signature yielding AUCs of 0.808, 0.785, and 0.778 for 1-, 3-, and 5-year survival, respectively (Fig. 6C and D). Bootstrapping validation (1000-iterations) yielded mean AUCs ± SD of 0.78 ± 0.05, 0.79 ± 0.04, and 0.76 ± 0.03 for 1-, 3-, and 5-year OS, respectively. Slight discrepancy with the original point estimated AUCs resulted from random sampling variation during resampling. The 3- and 5-year mean AUCs were highly consistent with original values, and SDs ≤0.04 confirmed robust internal validity and low overfitting risk. The larger 1-year AUC SD (0.055) was associated with limited 1-year survival events, increasing sensitivity to sampling variation.

These results demonstrate that the 17-gene signature has high sensitivity and specificity for predicting OS. To further evaluate the independent prognostic value of the 17-gene signature beyond the WHO molecular classification, the authors performed Kaplan-Meier survival analysis stratified by each molecular subgroup (WNT, SHH, Group 3, Group 4) in the entire cohort (Supplementary Fig. 1). The results showed that the high-risk group had significantly poorer prognosis than the low-risk group in all subgroups except WNT, with the most pronounced difference observed in Group 4.

In the test dataset, the high-risk group also showed significantly worse prognosis compared to the low-risk group (*p* = 0.0027), with corresponding AUCs of 0.785, 0.648, and 0.648 for 1-, 3-, and 5-year survival (Fig. 6E and F). In the RNA-seq validation set, exploratory findings consistent with the training and test sets were observed: the high-risk group demonstrated inferior clinical outcomes (*p* = 0.032) (Fig. 6G‒H). Due to the small sample size (*n* = 37) and limited 5-year follow-up data, only 1- and 3-year survival were analyzed, with exploratory AUCs of 0.786 and 0.783, respectively (Fig. 6I). These exploratory results provide preliminary clues regarding the potential prognostic value of the 17-gene signature in MB, but should be interpreted with caution given the constraints of the small external validation cohort.

### Independent prognostic value of the prognostic gene signature

In our study, the risk score is an independent predictor of overall survival (*p* < 0.001) (Supplementary Fig. 2). A training cohort of 400 patients, both univariate and multivariate Cox regression analyses validated the prognostic model as an independent predictor of overall survival (Supplementary Fig. 2A‒B). In the test cohort, both univariate and multivariate Cox regression analyses confirmed the model as an independent prognostic factor for overall survival (Supplementary Fig. 2C‒D). These findings indicate that the prognostic model can effectively predict OS in MB patients.

### Development and validation of a predictive nomogram

To create a clinically applicable method for predicting the survival probabilities of MB patients, a nomogram was developed using the training cohort. All independent prognostic parameters and relevant clinical variables were incorporated into the construction of this prognostic nomogram through a stepwise Cox regression model, allowing for the prediction of 1-, 3-, and 5-year OS in MB patients (Supplementary Fig. 3).

### CCK-8 cell proliferation assay

**Fig. 7:**
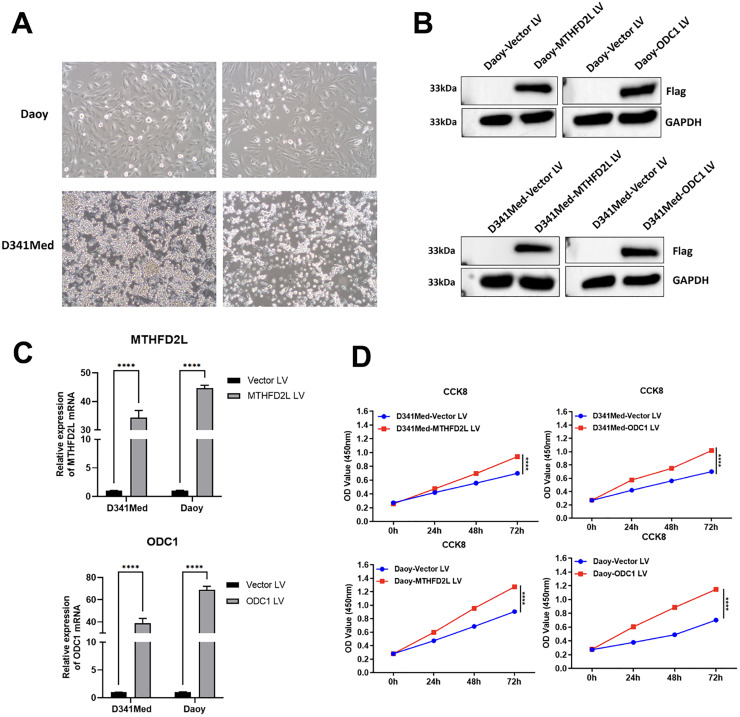
Over ‒ expression of MTHFD2L and ODC1 genes promotes cell proliferation. (A) It shows that the Daoy and D341Med cell lines were successfully resuscitated and grew well; (B) A protein electrophoresis image detecting the protein expression in the Daoy and D341Med cell lines after transfection with different lentiviruses (MTHFD2L LV, ODC1 LV), using Flag as a marker and GAPDH as an internal reference. The results indicate successful lentiviral transfection; (C) The relative transcriptional levels of the target genes (MTHFD2L, ODC1) in the Vector LV group, MTHFD2L LV group, and ODC1 LV group in the Daoy and D341Med cell lines were detected by PCR. It can be observed that both genes are highly expressed in the two cell lines; (D) The result image of the CCK8 cell proliferation assay, presenting the cell proliferation of the Vector LV group, MTHFD2L LV group, and ODC1 LV group in the Daoy and D341Med cell lines at different time points (0 h, 24 h, 48 h, 72 h).

To further investigate the potential mechanism by which the 17-gene model predicts medulloblastoma prognosis, cell proliferation experiments were performed on two genes ‒ MTHFD2L and ODC1 (both highly expressed in medulloblastoma) ‒ that contribute significantly to the risk score, using Daoy and D341Med cell lines. First, overexpression lentiviruses for the two target genes were constructed, and the successful construction of the target plasmids was verified via first-generation sequencing. Next, the two cell lines were resuscitated and subcultured. Upon reaching the required quantity for the experiment, cells were counted, and their viability was assessed. When cell viability reached 90 %, cells were seeded for transfection (Fig. 7A). Successful transfection was confirmed via protein electrophoresis (Fig. 7B), and PCR verification showed that target gene expression was significantly higher in all Overexpression (OE) groups compared with the Vector group (Fig. 7C). Results showed that overexpression of MTHFD2L and ODC1 significantly enhanced cell proliferation ability compared with the Vector group (Fig. 7D), consistent with our previous observations.

### Transwell migration assay

**Fig. 8:**
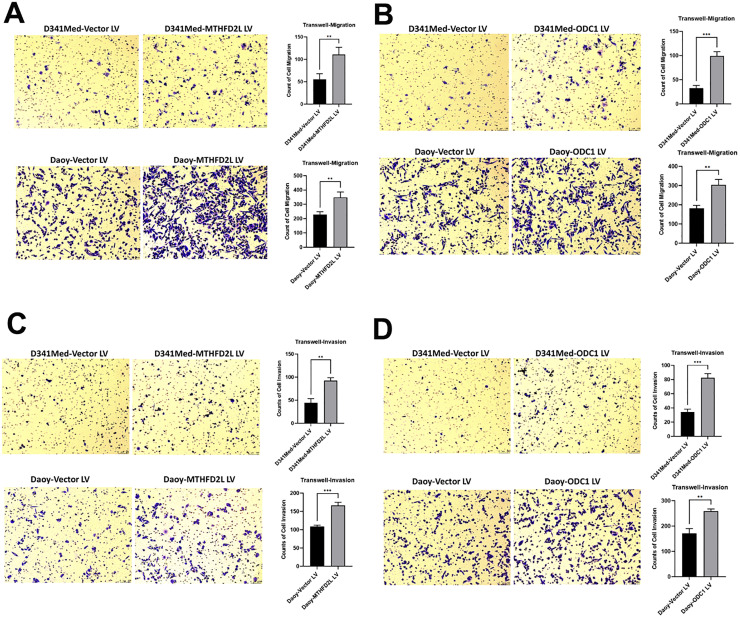
Over ‒ expression of MTHFD2L and ODC1 can enhance the migration and invasion abilities of tumor cells. (A) Compared with the Vector group, the migration ability of tumor cells is significantly increased after over ‒ expressing MTHFD2L; (B) Compared with the Vector group, the migration ability of tumor cells is significantly increased after over ‒ expressing ODC1; (C) Compared with the Vector group, the invasion ability of tumor cells is significantly increased after over ‒ expressing MTHFD2L; (D) Compared with the Vector group, the invasion ability of tumor cells is significantly increased after over ‒ expressing ODC1.

To clarify the effects of MTHFD2L and ODC1 overexpression on the migration and invasion abilities of tumor cells, cell migration and invasion assays were performed. Migration ability: As shown in Fig. 8A and B, compared with the empty vector-transfected control group (Vector LV), the number of cells passing through the micropores increased significantly in the overexpression groups ‒ D341Med-MTHFD2L LV, Daoy-MTHFD2L LV, D341Med-ODC1 LV, and Daoy-ODC1 LV. Statistical bar charts further confirmed significant differences, indicating that overexpression of both genes markedly enhances tumor cell migration.

Invasion ability: As depicted in Fig. 8C and D, compared with the Vector LV control group, overexpression of MTHFD2L or ODC1 significantly increased the number of cells passing through Matrigel-coated micropores. Statistical bar charts validated that overexpression of both genes notably promotes tumor cell invasion.

In summary, overexpression of MTHFD2L and ODC1 significantly enhances the migration and invasion abilities of D341Med and Daoy tumor cells.

## Discussion

MB is a common malignant tumor of the central nervous system in children with a poor prognosis. Currently, the mechanisms underlying MB development remain controversial. Histopathologically, MB is classified into four subgroups, each involving distinct gene mutations. With the advancement of molecular subtyping, genetic testing has become pivotal for MB classification and treatment. Metabolic reprogramming is a key feature of cancerous malignant transformation and plays a critical role in tumor pathophysiological processes. Thus, personalized treatments based on specific metabolic biomarkers may improve prognosis. Molecular prognostic markers, which can be measured via standardized detection techniques, evolve dynamically alongside disease progression and reflect patient prognosis. Given the challenge of tumor heterogeneity, a panel of molecular markers is likely to predict MB prognosis more accurately than any single marker alone.

In this study, an analysis of two mRNA datasets was performed to identify the metabolic subclasses of MB. Results revealed that MB can be categorized into four distinct metabolism-associated subclasses (C1, C2, C3, and C4), which was validated in the test set. Each subclass was associated with distinct clinical subtypes and involved different DEGs, GO terms, and KEGG pathways. Three mRNA microarray datasets were further analyzed to develop a metabolism-associated signature with robust prognostic predictive performance in MB. This signature consisted of 17 metabolic genes. These genes were not only differentially expressed between MB and healthy brain tissues but also significantly correlated with the OS of MB patients. In both the training and test sets, a high-risk score was linked to a notably poorer prognosis.

It is important to emphasize that these four metabolic subclasses are not intended to replace the established WHO molecular subgrouping system, but rather to serve as a metabolic refinement of existing subtypes. This refinement uncovers subtype-specific metabolic vulnerabilities that are not captured by conventional molecular typing, thereby providing actionable biological insights and therapeutic directions beyond current classification frameworks. In detail, the results showed that C1 contains the most different metabolic pathways; 21 pathways were upregulated, and 4 were related to amino acid metabolism, including homocysteine biosynthesis, cysteine and methionine metabolism, methionine cycle, and selenium compound metabolism. Methionine, which is an essential amino acid in mammals, is related to many important metabolic pathways that play key roles in polyamine synthesis, glutathione formation, and methyl group donation.^15^, ^16^, ^17^, ^18^ Many tumor cells show methionine dependency, which is considered to be a metabolic defect, while normal tissues can utilize homocysteine to fulfill their methionine requirements.^19^^,^^20^ This defect is linked to methionine synthase's failure to recycle homocysteine in rapidly dividing cells. The cause is complex and may involve altered methylation patterns in tumor DNA, affecting gene expression.^21^ Methionine restriction may therefore offer a targeted therapeutic strategy for C1 MB, potentially sparing normal tissues from off-target effects. Moreover, clinical feature analyses revealed that the majority of C1 samples belonged to the WNT subtype, and methionine restriction therapy may be equally applicable to this subtype.

C3 was also a metabolically active subtype, with 15 upregulated pathways, of which 4 were related to carbohydrate metabolism, including pentose phosphate, glyoxylate and dicarboxylate metabolism, butanoate metabolism and propanoate metabolism; the other 3 were related to lipid metabolism. Metabolic patterns commonly observed in cancer include increased lipogenesis and carbohydrate metabolism.^22^^,^^23^ In MB, activation of the SHH pathway induces a characteristic metabolism in cerebellar granule neuron progenitors, which involves decreased fatty acid oxidation, aerobic glycolysis and increased lipogenesis.^24^, ^25^, ^26^ Clinically, C3 correlates highly with SHH, demonstrating that our results are consistent with previous studies. Although it is difficult to distinguish between Group 3 and Group 4 subtypes in clinical practice, the differences between the groups with regard to metabolic grouping are significant.

For C4, only one significantly different metabolic pathway was identified, indicating no obvious metabolic activity. However, its clinical value lies in prognostic refinement for Group 4 (the most heterogeneous WHO subgroup), which accounts for the largest proportion of C4 samples (Supplementary Table 2). Conventional molecular typing fails to distinguish high-risk from low-risk Group 4 patients, but our 17-gene signature effectively stratifies this subgroup, highlighting C4’s role as a metabolic refinement for clinically challenging subtypes.

The four metabolism-associated MB subclasses identified in this study provide a metabolic perspective for interpreting the traditional WHO molecular classification, and may offer preliminary clinical translational insights for personalized MB treatment. For metabolically active C1 and C3 subclasses, targeted inhibition of abnormally activated metabolic pathways could be considered as a potential therapeutic option; for metabolically hypoactive C2 subclass, metabolism-targeted therapies may be less recommended to reduce unnecessary clinical interventions; and the 17-gene metabolic signature may assist in prognostic stratification for C4 subclass with no obvious metabolic characteristics, which might help optimize individualized treatment plans for highly heterogeneous Group 4 patients. This metabolic exploration of MB subtypes may complement the clinical application of traditional molecular typing to some extent and lay a preliminary theoretical foundation for the subsequent development of metabolism-targeted therapeutic strategies for MB. The authors then developed a metabolism-associated gene signature based on the above results, which exhibited superior performance in predicting MB prognosis. This signature comprises 17 metabolic genes: MTHFD2L, QDPR, ODC1, GRIA3, TTYH1, RRM2, ATP2B3, HS3ST1, and GRIA1 were upregulated and identified as negative genes, while ANKH, SLC24A3, DSE, ATP8A2, ATP6V1G2, GAD1, NALCN, and KCNK1 were downregulated and associated with good survival. The majority of these 17 signature genes participate in neurological functions, tumor proliferation, and tumor migration. For instance, MTHFD2L, an isoenzyme in the mitochondrial folate pathway, is highly expressed across multiple cancer types and facilitates tumor cell proliferation.^27^^,^^28^ QDPR, an enzyme participating in tetrahydrobiopterin metabolism, also acts to repair oxidative damage to tetrahydrofolate and is upregulated in breast cancer.^28^^,^^29^ ODC1 plays a crucial role in neurodevelopmental disorders and brain development.^30^ ODC1 also promotes proliferation and mobility via the AKT/GSK3β/β-catenin pathway and modulation of the acidotic microenvironment in human hepatocellular carcinoma.^31^ By activating the MAPK/ERK signaling pathway, GRIA3 promotes cell invasion and metastasis in non-small cell lung cancer.^32^ TTYH1 regulates Volume-Regulated Anion Channels (VRACs) and is involved in cellular functions such as regulation of cell volume, proliferation, and migration.^33^^,^^34^ RRM2 promotes non-small cell lung cancer progression by targeting the WNT axis.^35^ HS3ST1 is highly expressed in innate lymphoid cells of late-stage colorectal cancer, and the deficiency of HS3ST1 or PD1 in innate lymphoid cells suppresses tumor growth.^36^ GRIA1 has an important function in the treatment of leukemia.^37^

In this study, cell function experiments further validated the biological functions of MTHFD2L and ODC1 in the 17-gene model, providing experimental evidence for the potential mechanism by which this model predicts medulloblastoma prognosis. To clarify the scope of the functional assays performed herein, the authors note that this validation focuses on the universal oncogenic mechanisms of medulloblastoma, rather than subtype-specific biological processes. Mechanistically, MTHFD2L, as a key isoenzyme in the mitochondrial folate pathway, may provide a material basis for rapid proliferation by enhancing folate metabolism and one-carbon unit supply in tumor cells through its high expression. Notably, MTHFD2L is specifically enriched in the C1 subclass (WNT-associated), which exhibits prominent methionine metabolism activation (Supplementary Table 5). Previous studies have demonstrated that methionine metabolism regulates WNT pathway activation via DNA methylation ‒ methionine-derived S-Adenosylmethionine (SAM) serves as a key methyl donor to modulate the methylation status of WNT pathway regulators, thereby promoting downstream signaling. Our functional data demonstrating that MTHFD2L overexpression enhances the proliferative capacity of C1 subtype cells, together with previous reports confirming that methionine metabolism modulates WNT pathway activation via DNA methylation-mediated regulation of pathway regulators, suggest that MTHFD2L may promote MB progression in the C1 subclass through a methionine metabolism-dependent WNT pathway activation mechanism ‒ a hypothesis that warrants direct experimental validation in subsequent studies. This potential regulatory link provides a tentative explanation for how metabolic gene expression may couple with canonical oncogenic signals to drive subtype-specific tumorigenesis in MB.

ODC1, as a rate-limiting enzyme in polyamine synthesis, may be involved in the regulation of tumor cell growth by modulating intracellular polyamine levels, which is consistent with the conclusion from existing studies that metabolic reprogramming drives malignant phenotypes of tumors. ODC1 is predominantly associated with the C3 subclass (SHH-associated), characterized by activated carbohydrate and lipid metabolism (Supplementary Table 5). Accumulating evidence indicates that polyamine metabolism interacts with the SHH pathway to drive MB tumorigenesis ‒ polyamines can stabilize Gli1 (a core transcription factor of the SHH pathway) by inhibiting its ubiquitin-dependent degradation, sustaining pathway activation. Our clinical feature analysis establishing a strong correlation between the C3 subclass and the SHH molecular subtype, in conjunction with published evidence that polyamine metabolism stabilizes Gli1 to sustain SHH pathway activation in MB, indicates that ODC1 may exert its oncogenic effects in C3/SHH subtype MB through a polyamine metabolism-mediated SHH pathway activation mechanism, which remains to be verified by direct experimental tests. This potential crosstalk between metabolic and oncogenic signaling pathways highlights a possible molecular mechanism underlying the progression of C3/SHH subtype MB, and its clinical relevance requires further investigation.

### Limitation

A notable limitation of our current study is the relatively small sample size (*n* = 37) in the external validation cohort, which may restrict the generalizability of our 17-gene prognostic model and increase the risk of overfitting. Additionally, due to the scarcity of large-scale, publicly available medulloblastoma transcriptomic datasets with complete prognostic information, our model development and initial validation relied on subsets of the same primary dataset (GSE85217), which could introduce biases. These constraints highlight the need for further validation in larger, independent cohorts to confirm the robustness and clinical utility of the proposed signature. The authors are actively engaged in multi-center collaborations to accumulate additional samples, with the goal of addressing this limitation in future studies.

Furthermore, although the authors focused on metabolism-associated genes and their functional implications, the lack of dedicated human medulloblastoma metabolomics datasets (e.g., metabolite profiling data) limited our ability to directly validate the correlation between gene expression and metabolite levels. Future studies will integrate targeted metabolomics experiments to measure metabolite changes corresponding to the identified metabolic genes, thereby deepening the understanding of their roles in MB pathophysiology.

Additionally, although the authors have experimentally validated the core genes with the most prominent prognostic effects from the 17-gene signature, the functional roles of the remaining 15-genes in MB initiation and progression have not been explored and lack experimental validation, which will be the focus of our subsequent mechanistic research. On the other side, this study is based on retrospective data and lacks prospective validation, which may limit the clinical applicability of our findings.

## Conclusion

This study investigates the classification of Medulloblastoma (MB) based on metabolic signatures, thereby supplementing existing research on MB subtype characterization from a metabolic perspective. Our research provides preliminary insights into the metabolic hallmarks of MB and offers a potential reference for the subsequent development of multimolecule-based personalized therapeutic strategies and prognostic prediction tools ‒ with the caveat that the 17-gene signature requires additional multi-center validation to confirm its clinical utility.

## Ethics approval and consent to participate

This study was conducted in accordance with the Declaration of Helsinki. This study was conducted with approval from the Ethics Committee of Beijing Tiantan Hospital, Capital Medical University (n° KY2022–082–01). Written informed consent was obtained from the participants. This study follows the STROBE Statement.

## Consent for publication

Written informed consent was obtained from the participants.

## Data availability

The datasets used and/or analyzed during the current study were publicly available from the GEO database, http://www.ncbi.nlm.nih.gov/geo. Analysis scripts used in the current study are available from the corresponding author on reasonable request.

## Funding

This work was supported by a grant from the Institute of Artificial Intelligence, Hefei Comprehensive 10.13039/100017085National Science Center (grant number 21KT012) and the 10.13039/501100001809National Natural Science Foundation of China (Grant n°62276027).

## CRediT authorship contribution statement

**Zihan Yan:** Data curation, Formal analysis, Writing – original draft. **Yunwei Ou:** Data curation, Formal analysis. **Xu Han:** Data curation, Formal analysis. **Jian Gong:** Conceptualization, Writing – review & editing, Supervision.

## Declaration of competing interest

The authors declare no conflicts of interest.

## Appendix Supplementary materials

Image, application 1

Image, application 2

Image, application 3

Image, application 4

## Footnotes

Supplementary material associated with this article can be found, in the online version, at doi:10.1016/j.clinsp.2026.101003.
