# Supplementary material for: Identification of metabolism-associated molecular classification and prognostic genes for medulloblastoma based on bioinformatics analysis
Source: Clinics (Sao Paulo). 2026 May 18;81:101003. doi: 10.1016/j.clinsp.2026.101003 (PMC13202577; doi:10.1016/j.clinsp.2026.101003)

CLINICS-D-25-01337_Supplementary Material

**Supplementary Table 1** Details of the GEO datasets included in this study.

| **Datasets** | **Reference** | **Platform** | **Sample size (tumor/control)** |
| --- | --- | --- | --- |
| GSE50161 | Griesinger et al. (2013) | GPL570 | 35 (22/13) |
| GSE74195 | de Bont et al. (2008) | GPL570 | 28 (23/5) |
| GSE86574 | Amani et al. (2017) | GPL570 | 26 (16/10) |
| GSE85217 | Cavalli et al. (2017) | GPL22286 | 599 (599/0) |
| GSE37418 | Giles Robinson et al. (2012) | GPL570 | 74 (74/0) |

**Supplementary Table 2** Clinicopathological characteristics of patients included in this study.

| **ID** | **Molecular Subtype** | **Age** | **Gender** | **OS day** | **OS year** | **Survival-status** | **Eisk** | **Coef** |
| --- | --- | --- | --- | --- | --- | --- | --- | --- |
| TG1 | WNT | 8 | F | 1341 | 3.673973 | Y | Low | -1378.63 |
| TG10 | G4 | 13 | M | 1315 | 3.60274 | Y | Low | -914.768 |
| TG11 | G3 | 5 | F | 896 | 2.454795 | N | High | 375.8381 |
| TG12 | G3 | 6 | M | 790 | 2.164384 | N | High | 623.496 |
| TG13 | G4 | 7 | F | 1246 | 3.413699 | Y | High | 256.2029 |
| TG14 | G4 | 9 | M | 1225 | 3.356164 | Y | High | 260.1781 |
| TG15 | SHH | 2 | M | 1224 | 3.353425 | Y | Low | 217.285 |
| TG18 | G4 | 6 | M | 1217 | 3.334247 | Y | High | 815.8478 |
| TG2 | G3 | 4 | F | 384 | 1.052055 | N | High | 628.5418 |
| TG20 | G3 | 2 | F | 1208 | 3.309589 | Y | High | 1035.761 |
| TG21 | SHH | 9 | M | 1207 | 3.306849 | Y | High | 962.2383 |
| TG22 | G4 | 13 | M | 1205 | 3.30137 | Y | Low | 73.70473 |
| TG23 | G4 | 7 | M | 1203 | 3.29589 | Y | High | 361.6017 |
| TG24 | G4 | 8 | M | 1217 | 3.334247 | Y | High | 758.7663 |
| TG26 | G4 | 3 | M | 1183 | 3.241096 | Y | Low | -50.0425 |
| TG27 | WNT | 6 | M | 1182 | 3.238356 | Y | Low | -805.205 |
| TG28 | WNT | 9 | F | 1179 | 3.230137 | Y | Low | -828.031 |
| TG29 | G4 | 8 | M | 1173 | 3.213699 | Y | Low | 189.7378 |
| TG3 | G4 | 15 | M | 1334 | 3.654795 | Y | Low | 23.82234 |
| TG30 | G4 | 5 | M | 1105 | 3.027397 | Y | Low | -399.792 |
| TG31 | G4 | 2 | M | 1105 | 3.027397 | Y | High | 1324.137 |
| TG32 | WNT | 7 | M | 1112 | 3.046575 | Y | Low | -1007.64 |
| TG33 | SHH | 12 | M | 1118 | 3.063014 | Y | High | 387.1448 |
| TG34 | G4 | 5 | M | 1123 | 3.076712 | Y | High | 447.7336 |
| TG35 | SHH | 2 | F | 1112 | 3.046575 | Y | High | 265.9618 |
| TG36 | SHH | 8 | F | 1113 | 3.049315 | Y | Low | 253.55 |
| TG37 | G4 | 7 | M | 1049 | 2.873973 | Y | Low | -328.347 |
| TG38 | G4 | 7 | M | 1047 | 2.868493 | Y | High | 794.7547 |
| TG39 | WNT | 4 | M | 1134 | 3.106849 | Y | Low | -480.459 |
| TG4 | G4 | 9 | F | 243 | 0.665753 | N | High | 1097.487 |
| TG40 | G4 | 15 | M | 1141 | 3.126027 | Y | High | 805.8612 |
| TG41 | G3 | 8 | M | 85 | 0.232877 | N | High | 295.2203 |
| TG5 | G4 | 6 | M | 1336 | 3.660274 | Y | Low | -733.363 |
| TG6 | G4 | 13 | M | 1300 | 3.561644 | Y | Low | -276.586 |
| TG7 | G3 | 7 | M | 1301 | 3.564384 | Y | Low | 74.29303 |
| TG8 | G3 | 10 | M | 1007 | 2.758904 | N | High | 679.8045 |
| TG9 | G3 | 4 | F | 1211 | 3.317808 | N | Low | -56.6871 |

**Supplementary Table 3** Univariate and multivariate Cox regression analysis of prognostic factors.

| **ID** | **Subtype** | **Cluster** | **Age** | **Gander** | **Survival Status** | **OS (months)** |
| --- | --- | --- | --- | --- | --- | --- |
| Y7 | G4 | C1 | 9 | M | Decease | 14 |
| Y4 | WNT | C1 | 14 | F | Decease | 41 |
| Y6 | G4 | C2 | 5 | F | Decease | 20 |
| Y1 | G4 | C2 | 7 | F | Decease | 15 |
| Y5 | SHH | C3 | 8 | F | Alive | 59 |
| Y3 | SHH | C3 | 11 | M | Decease | 39 |
| Y2 | SHH | C3 | 12 | M | Decease | 99 |
| Y8 | G3 | C4 | 8 | M | Alive | 50 |

**Supplementary Table 4** Metabolic genes differentially expressed among molecular subgroups.

[excel]

**Supplementary Table 5** Functional enrichment analysis of metabolic subclasses.

| **Differentially metabolism pathways in C1** | | | **Differentially metabolism pathways in C2** | | | **Differentially metabolism pathways in C3** | | | **Differentially metabolism pathways in C4** | | |
| --- | --- | --- | --- | --- | --- | --- | --- | --- | --- | --- | --- |
| **Pathway** | **logFC** | **adj.P.Val** | **Pathway** | **logFC** | **adj.P.Val** | **Pathway** | **logFC** | **adj.P.Val** | **Pathway** | **logFC** | **adj.P.Val** |
| Remethylation | 0,365167318 | 2,23E-25 | Other Types of O-Glycan Biosynthesis | 0,22520901 | 7,55E-32 | Glycogen Biosynthesis | 0,474435136 | 1,59E-54 | Glycogen Degradation | 0,221055 | 6,42E-12 |
| Purine Biosynthesis | 0,353906708 | 8,65E-16 | Glycolysis | -0,203709038 | 4,42E-55 | Purine Biosynthesis | 0,358146892 | 2,19E-37 |  |  |  |
| Epinephrine Biosynthesis | 0,347747528 | 9,05E-15 | Methionine Cycle | -0,205881538 | 1,58E-36 | Selenocompound Metabolism | 0,333447462 | 4,16E-37 |  |  |  |
| Neomycin, Kanamysin and Gentamicin Biosynthesis | 0,340944201 | 2,23E-12 | Cysteine and Methionine Metabolism | -0,212085953 | 5,87E-38 | Glyoxylate and Dicarboxylate Metabolism | 0,324210929 | 1,20E-53 |  |  |  |
| Sulfur Metabolism | 0,336857324 | 2,25E-19 | Prostaglandin Biosynthesis | -0,213635084 | 1,07E-16 | Retinoic Acid Metabolism | 0,3154883 | 8,09E-35 |  |  |  |
| Homocysteine Biosynthesis | 0,33241013 | 4,03E-16 | Mucin Type O-Glycan Biosynthesis | -0,218052418 | 1,85E-38 | Pentose Phosphate | 0,313060909 | 4,39E-51 |  |  |  |
| Glycogen Biosynthesis | 0,325062094 | 6,15E-11 | Retinoic Acid Metabolism | -0,221906011 | 5,29E-20 | Steroid Biosynthesis | 0,274037414 | 9,54E-29 |  |  |  |
| Norepinephrine Biosynthesis | 0,303151579 | 3,23E-10 | Pyrimidine Biosynthesis | -0,231018313 | 1,16E-17 | Folate One Carbon Metabolism | 0,248687224 | 6,25E-31 |  |  |  |
| Glycogen Degradation | 0,281541338 | 6,79E-12 | Glyoxylate and Dicarboxylate Metabolism | -0,232247417 | 6,27E-31 | Nicotinamide Adenine Dinucleotide Biosynthesis | 0,239293611 | 4,81E-13 |  |  |  |
| Prostaglandin Biosynthesis | 0,276458689 | 8,70E-11 | Remethylation | -0,247220758 | 1,44E-31 | Ketone Biosynthesis and Metabolism | 0,233302582 | 4,23E-21 |  |  |  |
| Glycosaminoglycan Biosynthesis | 0,274814355 | 3,37E-30 | Ketone Biosynthesis and Metabolism | -0,249271376 | 6,04E-29 | Prostaglandin Biosynthesis | 0,224729652 | 1,83E-15 |  |  |  |
| Cysteine and Methionine Metabolism | 0,268158664 | 7,10E-22 | Steroid Biosynthesis | -0,260835357 | 4,68E-31 | Pyrimidine Biosynthesis | 0,22227534 | 8,10E-14 |  |  |  |
| Nicotinamide Adenine Dinucleotide Biosynthesis | 0,25706177 | 3,34E-07 | Folate One Carbon Metabolism | -0,285479034 | 6,66E-51 | Glycosphingolipid Biosynthesis | 0,220142964 | 3,26E-44 |  |  |  |
| Ketone Biosynthesis and Metabolism | 0,251112226 | 3,58E-11 | Pentose Phosphate | -0,286733114 | 6,66E-51 | Butanoate Metabolism | 0,219803782 | 8,16E-33 |  |  |  |
| Folate One Carbon Metabolism | 0,247985253 | 1,57E-13 | Homocysteine Biosynthesis | -0,314699274 | 2,43E-40 | Propanoate Metabolism | 0,201849554 | 1,46E-22 |  |  |  |
| Pentose Phosphate | 0,2354807 | 2,72E-12 | Purine Biosynthesis | -0,343346834 | 2,59E-41 | Arginine and Proline Metabolism | -0,217661516 | 2,38E-34 |  |  |  |
| Methionine Cycle | 0,227505453 | 4,03E-16 | Selenocompound Metabolism | -0,359063301 | 2,41E-53 | Fatty Acid Biosynthesis | -0,225490843 | 1,18E-41 |  |  |  |
| Urea Cycle | 0,222639107 | 1,75E-12 | Glycogen Biosynthesis | -0,385869642 | 1,72E-41 | Glycosphosphatidylinositol | -0,243876787 | 3,17E-35 |  |  |  |
| Selenocompound Metabolism | 0,218741786 | 1,96E-07 |  |  |  | Retinoid Metabolism | -0,515875665 | 1,22E-42 |  |  |  |
| Mucin Type O-Glycan Biosynthesis | 0,204334872 | 2,07E-12 |  |  |  |  |  |  |  |  |  |
| Steroid Biosynthesis | 0,203002791 | 1,93E-07 |  |  |  |  |  |  |  |  |  |
| Other Types of O-Glycan Biosynthesis | -0,264459123 | 4,03E-16 |  |  |  |  |  |  |  |  |  |

**Supplementary Table 6** Single-cell sequencing data processing and quality control.

[excel]

**Supplementary Table 7** Key metabolic pathway activity scores across cell clusters.

| **ID** | **p_val** | **avg_log2FC** | **pct.1** | **pct.2** | **p_val_adj** | **Cluster** | **Gene** |
| --- | --- | --- | --- | --- | --- | --- | --- |
| ATP1A2 | 0 | 2.128365 | 0.232 | 0.081 | 0 | C1 | ATP1A2 |
| GRIK2 | 9.9E-35 | 0.800617 | 0.271 | 0.226 | 2.28E-31 | C1 | GRIK2 |
| KCNJ6 | 0 | 2.597951 | 0.409 | 0.06 | 0 | C3 | KCNJ6 |
| GRIA4 | 0 | 2.169276 | 0.303 | 0.039 | 0 | C3 | GRIA4 |
| BCAT1 | 0 | 2.019461 | 0.394 | 0.092 | 0 | C3 | BCAT1 |
| LDHB | 0 | 2.017339 | 0.721 | 0.283 | 0 | C3 | LDHB |
| ADCY8 | 0 | 1.763738 | 0.251 | 0.058 | 0 | C3 | ADCY8 |
| ABCC4 | 0 | 1.756695 | 0.264 | 0.057 | 0 | C3 | ABCC4 |
| ATIC | 0 | 1.577281 | 0.437 | 0.161 | 0 | C3 | ATIC |
| DAGLA | 0 | 1.429621 | 0.255 | 0.07 | 0 | C3 | DAGLA |
| SLC6A15 | 0 | 1.340414 | 0.377 | 0.114 | 0 | C3 | SLC6A15 |
| ST8SIA1 | 0 | 1.312467 | 0.234 | 0.095 | 0 | C3 | ST8SIA1 |
| SLC1A5 | 0 | 0.838964 | 0.282 | 0.112 | 0 | C3 | SLC1A5 |
| FDFT1 | 3.7E-252 | 0.671635 | 0.296 | 0.163 | 8.6E-249 | C3 | FDFT1 |
| BDH1 | 2.4E-223 | 0.589439 | 0.278 | 0.155 | 5.6E-220 | C3 | BDH1 |
| ATP2B4 | 3.3E-161 | 0.404279 | 0.385 | 0.259 | 7.5E-158 | C3 | ATP2B4 |
| CA4 | 0 | 2.462984 | 0.338 | 0.055 | 0 | C2 | CA4 |
| KCNA5 | 0 | 2.254683 | 0.414 | 0.125 | 0 | C2 | KCNA5 |
| KCNA1 | 0 | 2.191737 | 0.272 | 0.043 | 0 | C2 | KCNA1 |
| GLRA1 | 0 | 2.029115 | 0.304 | 0.082 | 0 | C2 | GLRA1 |
| DGKB | 0 | 1.783562 | 0.271 | 0.094 | 0 | C2 | DGKB |
| ABCC8 | 0 | 1.554835 | 0.287 | 0.111 | 0 | C2 | ABCC8 |
| INPP5F | 0 | 1.525382 | 0.407 | 0.196 | 0 | C2 | INPP5F |
| ASS1 | 0 | 1.515868 | 0.261 | 0.12 | 0 | C2 | ASS1 |
| PDE1C | 0 | 1.501881 | 0.246 | 0.091 | 0 | C2 | PDE1C |
| DPYSL4 | 0 | 1.374886 | 0.596 | 0.396 | 0 | C2 | DPYSL4 |
| ADCY1 | 0 | 1.283728 | 0.545 | 0.33 | 0 | C2 | ADCY1 |
| B4GALT6 | 1.3E-306 | 1.237556 | 0.203 | 0.088 | 3E-303 | C2 | B4GALT6 |
| SLC38A1 | 0 | 1.190279 | 0.409 | 0.201 | 0 | C2 | SLC38A1 |
| CACNA1H | 3.6E-255 | 0.990745 | 0.207 | 0.099 | 8.4E-252 | C2 | CACNA1H |
| SV2B | 0 | 0.872096 | 0.364 | 0.207 | 0 | C2 | SV2B |
| CLCN4 | 4.4E-160 | 0.775993 | 0.308 | 0.207 | 1E-156 | C2 | CLCN4 |
| B3GAT1 | 1.47E-69 | 0.755937 | 0.258 | 0.201 | 3.38E-66 | C2 | B3GAT1 |
| CACNG2 | 3.42E-78 | 0.565 | 0.235 | 0.168 | 7.87E-75 | C2 | CACNG2 |
| CHST1 | 1.56E-60 | 0.487179 | 0.229 | 0.171 | 3.58E-57 | C2 | CHST1 |
| NPR3 | 0 | 4.848729 | 0.563 | 0.017 | 0 | C4 | NPR3 |
| GABRA5 | 0 | 3.891001 | 0.558 | 0.044 | 0 | C4 | GABRA5 |
| GRIA1 | 0 | 3.415903 | 0.448 | 0.047 | 0 | C4 | GRIA1 |
| SLC1A7 | 0 | 2.851731 | 0.514 | 0.051 | 0 | C4 | SLC1A7 |
| SLC12A2 | 0 | 1.631638 | 0.363 | 0.12 | 0 | C4 | SLC12A2 |

**Supplementary Table 8** Correlation analysis between 17-gene signature and immune infiltration.

[excel]

**Supplementary Table 9** Prediction performance of the 17-gene signature in validation cohorts.

| **Gene** | **Beta** | **HR (95% CI for HR)** | **wald.test** | **p-value** |
| --- | --- | --- | --- | --- |
| DSE | -0.72 | 0.49 (0.36‒0.65) | 23 | 1.90E-06 |
| ATP8A2 | -0.25 | 0.78 (0.7‒0.87) | 21 | 4.90E-06 |
| MTHFD2L | 0.65 | 1.9 (1.4‒2.6) | 19 | 1.30E-05 |
| SCN8A | -0.8 | 0.45 (0.31‒0.65) | 17 | 2.90E-05 |
| CACNA1A | -0.17 | 0.84 (0.78‒0.92) | 17 | 4.20E-05 |
| ODC1 | 0.6 | 1.8 (1.4‒2.4) | 16 | 5.00E-05 |
| NALCN | -0.25 | 0.78 (0.68‒0.89) | 14 | 0.00022 |
| RRM2 | 0.38 | 1.5 (1.2‒1.8) | 14 | 0.00024 |
| SV2A | -0.26 | 0.77 (0.67‒0.89) | 13 | 0.00036 |
| KCNQ2 | -0.29 | 0.75 (0.63‒0.89) | 10 | 0.0014 |
| QDPR | 0.55 | 1.7 (1.2‒2.4) | 10 | 0.0016 |
| MGAT4A | -0.29 | 0.75 (0.62‒0.9) | 9.3 | 0.0023 |
| GAD1 | -0.16 | 0.85 (0.76‒0.95) | 8.8 | 0.0031 |
| SLC6A1 | -0.24 | 0.79 (0.67‒0.93) | 8.2 | 0.0042 |
| GABRD | -0.27 | 0.76 (0.64‒0.92) | 8 | 0.0047 |
| LBR | 0.45 | 1.6 (1.1‒2.2) | 7.5 | 0.0061 |
| KCNJ6 | -0.15 | 0.86 (0.77‒0.96) | 7.2 | 0.0072 |
| ATP2B3 | 0.14 | 1.1 (1‒1.3) | 6.7 | 0.0095 |
| SCN1B | -0.35 | 0.7 (0.54‒0.92) | 6.6 | 0.01 |
| ANKH | -0.48 | 0.62 (0.43‒0.9) | 6.4 | 0.012 |
| KCNK1 | -0.16 | 0.85 (0.75‒0.97) | 6.2 | 0.012 |
| CA11 | -0.24 | 0.79 (0.65‒0.95) | 6.2 | 0.013 |
| GRIA1 | 0.12 | 1.1 (1‒1.3) | 5.1 | 0.024 |
| GRIA3 | 0.2 | 1.2 (1‒1.5) | 4.8 | 0.028 |
| SLC8A2 | -0.22 | 0.8 (0.66‒0.98) | 4.6 | 0.031 |
| HS3ST1 | 0.28 | 1.3 (1‒1.7) | 4.4 | 0.037 |
| KCNK12 | -0.22 | 0.8 (0.65‒0.99) | 4.3 | 0.039 |
| ST8SIA5 | -0.16 | 0.85 (0.73‒0.99) | 4.2 | 0.04 |
| TTYH1 | 0.17 | 1.2 (1‒1.4) | 4.2 | 0.041 |
| SLC24A3 | -0.15 | 0.86 (0.73‒1) | 3.9 | 0.049 |
| ATP6V1G2 | -0.24 | 0.79 (0.62‒1) | 3.9 | 0.049 |

**Supplementary Table 10** The clinical characteristics of the training and test sets.

| **Clinical characteristics** | **Training_set (n=400)** | **Testing_set (n=199)** | **RNAseq_set (n=37)** | **p-value** |
| --- | --- | --- | --- | --- |
| Gender (female) | 131 (32.8%) | 69 (34.7%) | 10 (27.0%) | 1 |
| Age | 10.28 (±9.35) | 10.18 (±8.93) | 7.32 (±3.57) | 0.158 |
| Subgroup |  |  |  |  |
| WNT | 41.2 (10%) | 19 (9.5%) | 5 (13.5%) | 0.643 |
| SHH | 109 (27.3%) | 59 (29.7%) | 5 (13.5%) |  |
| Group3 | 74 (18.5%) | 37 (18.6%) | 8 (21.6%) |  |
| Group4 | 176 (44%) | 84 (42.2%) | 19 (51.4%) |  |

**Supplementary Table 11** List of primer sequences and reagents used in this study.

| **Gene** | **Coef** | **Mean Expression (Training Set)** | **SD (Training Set)** |
| --- | --- | --- | --- |
| MTHFD2L | 0.66159926 | 5.23 | 0.87 |
| QDPR | 0.412769783 | 4.67 | 0.79 |
| ODC1 | 0.165440545 | 4.89 | 0.72 |
| GRIA3 | 0.143323094 | 6.11 | 1.03 |
| TTYH1 | 0.112264197 | 5.76 | 0.91 |
| RRM2 | 0.084668675 | 4.92 | 0.84 |
| ATP2B3 | 0.07549509 | 5.38 | 0.89 |
| HS3ST1 | 0.071117614 | 5.12 | 0.78 |
| GRIA1 | 0.057104973 | 6.05 | 1.12 |
| KCNK1 | -0.009904217 | 5.89 | 0.97 |
| NALCN | -0.055988713 | 5.42 | 0.86 |
| GAD1 | -0.07036893 | 5.67 | 0.93 |
| ATP6V1G2 | -0.122811454 | 6.21 | 1.05 |
| ATP8A2 | -0.144362938 | 5.98 | 0.99 |
| DSE | -0.207984737 | 5.73 | 0.88 |
| SLC24A3 | -0.251598841 | 5.56 | 0.82 |
| ANKH | -0.254280922 | 5.91 | 0.94 |

**Supplementary Figure 1** Kaplan-Meier survival analysis of the 17-gene signature stratified by WHO molecular subgroups. The 17-gene risk score effectively stratified overall survival in SHH (p=1.12 × 10^-3^, Group 3 (p = 1.55 × 10^-3^), and Group 4 (p = 2.75 × 10^-5^) medulloblastoma subgroups, but not in the WNT subgroup (p = 0.386), consistent with the favorable prognosis and small sample size of WNT medulloblastoma. High-risk groups are shown in blue, and low-risk groups are shown in yellow. The number of patients at risk at each time point is listed below each survival curve.


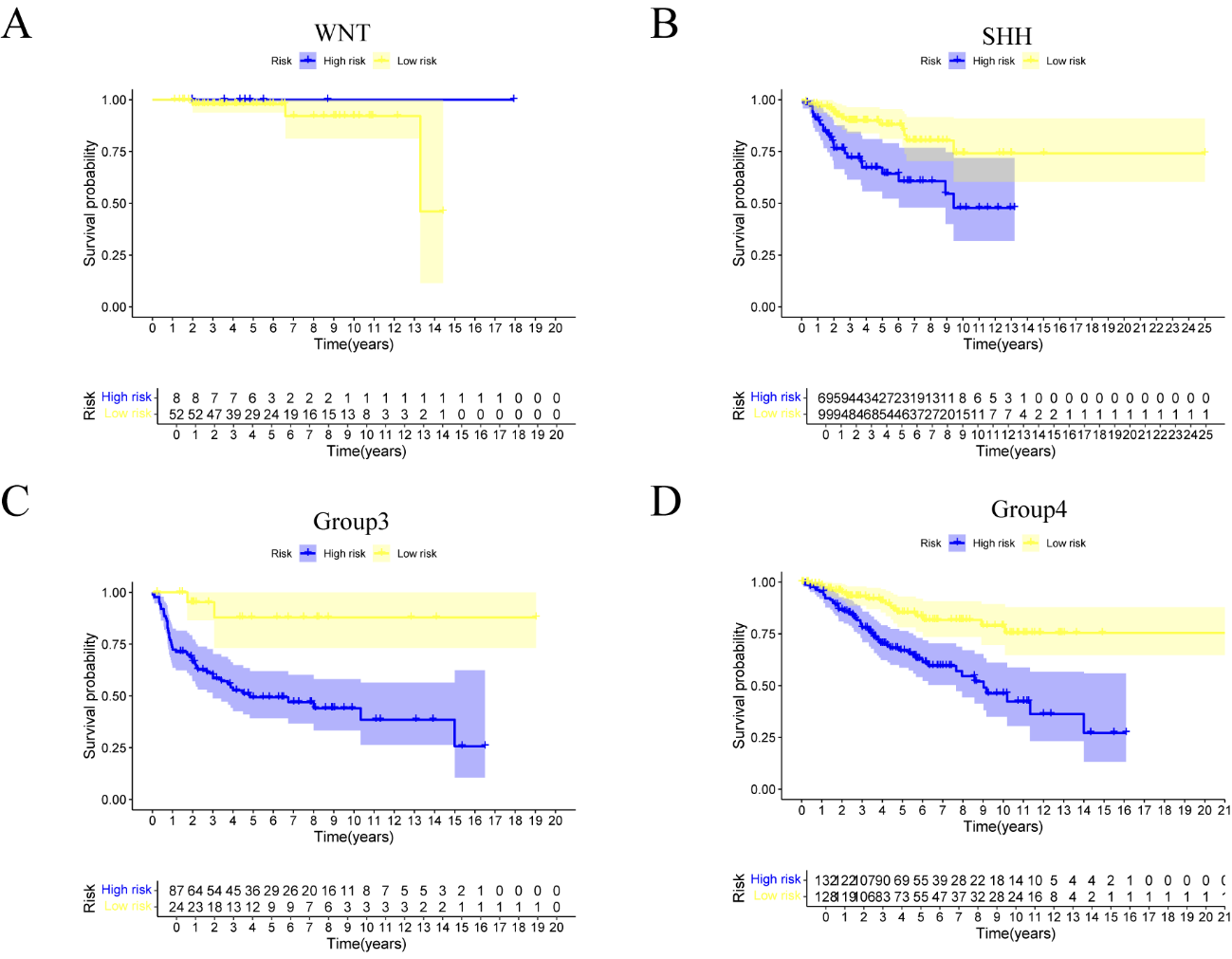


**Supplementary Figure 2** Cox regression analysis of the association between clinical factors and OS. (A‒B) Univariate and multivariate Cox regression analysis in train cohort; (C‒D) Univariate and multivariate Cox regression analysis in validation cohort.


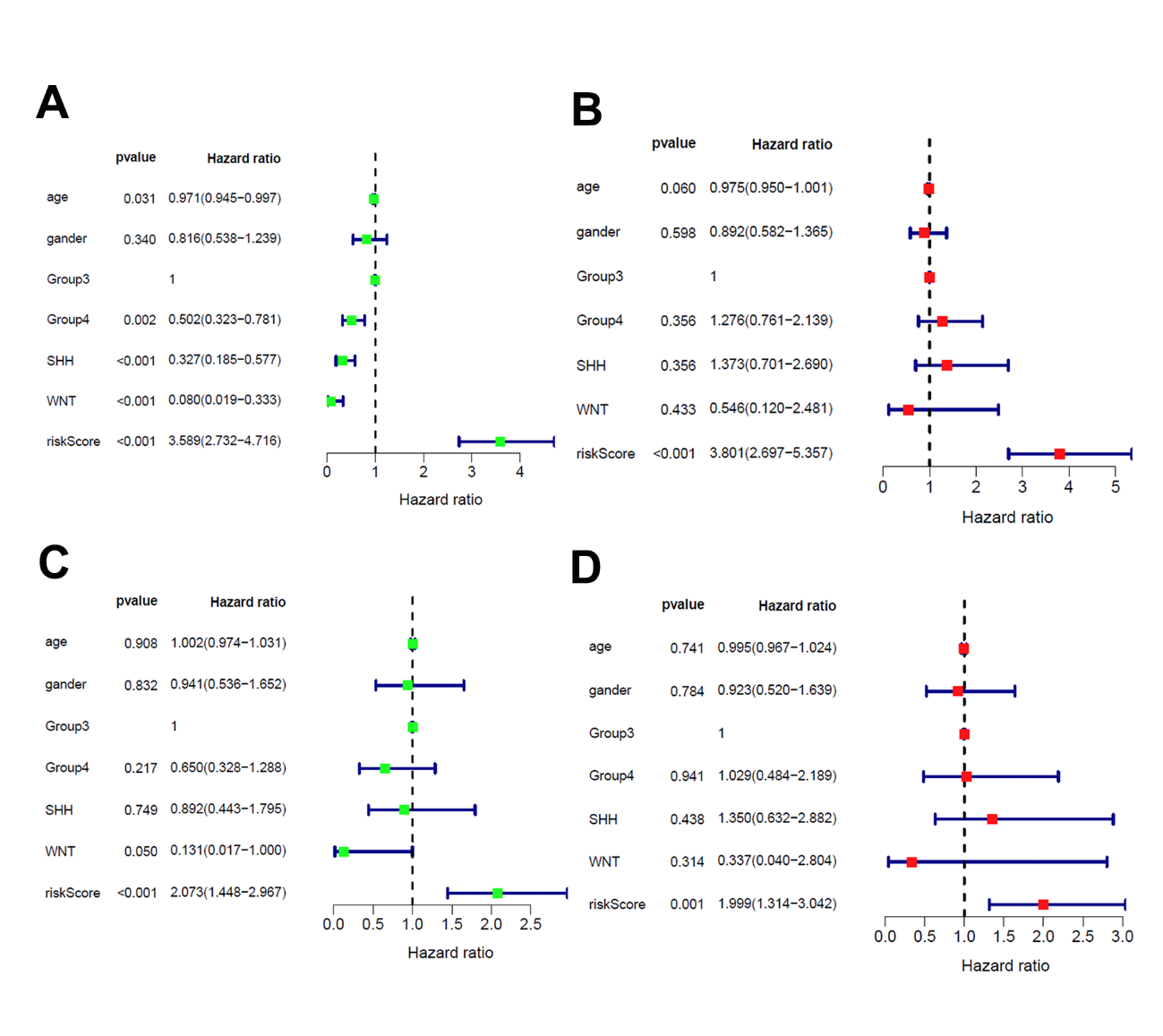


**Supplementary Figure 3** Nomogram combining 17-gene signature and clinical factors.


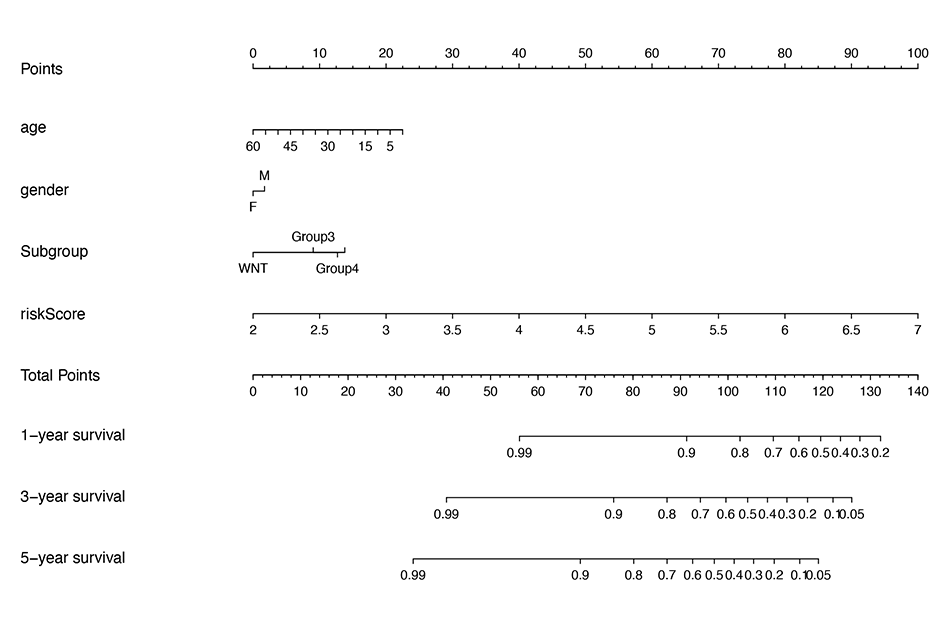

Supplement: Supplementary file 1 [file mmc1.docx]
